# War-Related Post-traumatic Stress Symptoms Among Healthcare Workers in Lebanon: A Cross-Sectional Study

**DOI:** 10.7759/cureus.100723

**Published:** 2026-01-04

**Authors:** Danielle Abou Khater, Ahmad Haj Hussein, Dana Awad, Fadi El Ters, Christeen Mina, Alain El Asmar

**Affiliations:** 1 Emergency Department, Lebanese American University Medical Center, Beirut, LBN; 2 Emergency Department, Lebanese American University School of Medicine, Beirut, LBN

**Keywords:** healthcare workers, lebanon, post-traumatic stress disorder, post-traumatic stress symptoms, war

## Abstract

Introduction

The 2023-2024 Israeli-Lebanese war has inflicted widespread trauma. On the front line, healthcare workers (HCWs) face psychological stressors, yet their mental health has received limited research. This study assesses the risk of post-traumatic stress symptoms (PTSS) development among Lebanese HCWs while exploring associations with sociodemographic and war-related factors.

Methods

A cross-sectional study was conducted at the Lebanese American University Medical Center-Rizk Hospital in Beirut, Lebanon, between December 2024 and June 2025. An anonymous, self-administered questionnaire was completed by 370 HCWs and included sociodemographic characteristics, war-related exposures, and the Post-traumatic Stress Disorder (PTSD) Checklist for Diagnostic and Statistical Manual of Mental Disorders, Fifth Edition (PCL-5). A PCL-5 score ≥33 indicated an elevated risk of PTSS. Statistical analyses included independent-samples t-tests, ANOVA or Welch’s ANOVA, and Pearson correlation, with non-parametric sensitivity analyses performed as appropriate.

Results

Among participants, 68 (18.4%) were at high PTSS risk. Higher PCL-5 scores were associated with female gender, the nursing profession, lower income, prior PTSD diagnosis, and direct war-related exposures, including displacement, personal injury, or loss of a relative. Income showed a significant negative correlation with PTSS severity. Among physicians, ophthalmologists exhibited the highest scores, correlating with the nature of injuries sustained during a mass bombardment on September 17, 2024.

Conclusion

Lebanese HCWs are at high risk for PTSD following the war, highlighting the urgent need for mental health interventions and support.

## Introduction

Lebanon is a small country situated along the Mediterranean Sea and extends over an area of 10,452 km² with a population of 6.7 million [1]. Bordered by Syria to the north and east, as well as Israel and the Palestinian territories to the south, Lebanon’s geopolitical location has made it a focal point for conflicts and military disputes over the past several decades [1-3]. Since 1978, Lebanon has witnessed a sequence of conflicts and invasions involving its southern neighbor, including the 1978 South Lebanon conflict, the Israeli military offensives in 1982, 1993, and 1996, not to mention the war between Israel and Lebanon in 2006 [4]. The most recent of these clashes involves the ongoing conflict with Israel that started on October 7, 2023, and escalated dramatically on September 23, 2024, with Israel’s bombardment of the Lebanese capital, Beirut. Israel’s invasion of southern Lebanon in response to resistance by the Lebanese armed group Hezbollah following the events of October 7 led to increasingly intensified Israeli airstrikes targeting cities in the South, the Bekaa Valley, and the southern suburbs of Beirut. Consequently, such events had a direct impact on tens of thousands of citizens, leading to mass casualties, widespread injuries, and a large-scale displacement as residents of the bombarded regions fled their homes in search of safety [5,6].

In addition to these challenges, Lebanon is grappling with a severe financial crisis, soaring rates of extreme poverty, and a critical shortage of healthcare resources and professionals. Electrical outages, reliance on private electrical generators, and the immigration of a large number of senior medical staff from the major hospitals of Lebanon have only added to the challenging medical environment during times of war [4]. The impact of ongoing warfare has further exacerbated the hardships faced by communities, healthcare systems, and providers alike [7]. Healthcare workers (HCWs), who are often on the frontlines of mass casualty incidents and pandemics, are frequently subjected to high-stress environments that can lead to serious mental health consequences. Prolonged exposure to such crises leaves them particularly vulnerable to post-traumatic stress disorder (PTSD), highlighting the profound psychological toll these repetitive conflicts impose on those frontline responders [8-10].

PTSD is recognized as the most common psychiatric outcome following disasters [11,12]. As defined by the Diagnostic and Statistical Manual of Mental Disorders, 5^th^ Edition (DSM-5), PTSD is a delayed and persistent mental health condition that develops at least one month after exposure to a life-threatening event, serious injury, natural disaster, or sexual violence. It is marked by recurrent intrusive thoughts, avoidance of trauma-related stimuli, negative alterations in mood and cognition, and heightened physiological arousal, manifesting as irritability, anger, sleep disturbances, and elevated heart rate or blood pressure [11]. Evidence suggests that early identification and intervention can effectively support the psychological well-being of those affected [11]. However, few studies have focused on the psychological impact of Lebanon’s repeated crises, particularly on HCWs [13,14].

The primary objective of this study is to evaluate the prevalence and severity of post-traumatic stress symptoms (PTSS) among Lebanese HCWs using the PTSD Checklist for DSM-5 (PCL-5), analyzed both as a continuous score and as a dichotomized outcome based on the validated cutoff of ≥33. The secondary objectives are to examine the associations between PCL-5 scores and predefined sociodemographic and occupational characteristics (such as age, gender, income, job profile, and medical specialty) and to assess the relationship between PTSS and direct war-related events, including displacement from residence, personal injury, or injury/loss of a relative or friend. These objectives guided the planned analytical framework, which incorporated group comparisons and correlation analyses to explore these relationships.

## Materials and methods

Study design

This cross-sectional, survey-based study was conducted between December 2024 and June 2025 at the Lebanese American University Medical Center-Rizk Hospital, Beirut, Lebanon. An electronic consent was sent to healthcare professionals, including physicians, fellows, residents, registered and practical nurses, pharmacists, and physiotherapists. Participation was entirely voluntary, and respondents had the right to withdraw their consent or discontinue their involvement at any time without consequence.

Sampling and data collection

A convenience sampling strategy was used. HCWs employed at the Lebanese American University Medical Center-Rizk Hospital during the study period were invited to participate by filling out a Google Form survey (Google Inc., Mountain View, CA) sent through official departmental WhatsApp groups (Meta Inc., Menlo Park, CA) and broadcast lists managed by unit supervisors. Approximately 612 HCWs were employed at the institution during the data collection period. Because WhatsApp does not indicate how many individuals viewed or received the survey link, the exact number of invited participants and, therefore, the precise response rate cannot be determined.

Participants were asked to complete the questionnaire anonymously. All participants provided informed consent after receiving an explanation of the survey’s nature and purpose. The questionnaire was designed in alignment with the study objectives and developed based on a thorough literature review.

Participants eligible for participation were individuals aged between 18 and 65 years, residing in Lebanon since the start of the war, who had provided their consent to participate in the survey. The participants were HCWs residing in Lebanon, working either directly during the incidents or indirectly with the repercussions of the tragic events. Non-medical staff were not included. The following groups of HCWs were assessed: physicians, fellows and residents, registered nurses, practical nurses, pharmacists, and physiotherapists.

The self-reported questionnaire was written in English, beginning with a brief introductory note outlining the study's objectives and assuring participants of the confidentiality and anonymity of their responses, followed by a consent form.

The questionnaire consisted of 35 questions divided into three sections: sociodemographic characteristics (age, gender, nationality, marital status, and place of residence prior to the events), job profile (profession, specialty, and monthly personal income), and mental health background (prior PTSD diagnosis). Questions about the direct and indirect implications of the ongoing war on the participants: Displacement as forced relocation from the participant’s primary residence due to the ongoing war, either temporarily or permanently; the war’s impact on the applicant’s mental health; sustaining personal injury; injury or loss of a relative; and presence at the workplace during Code White activation. Code White at the Lebanese American University Medical Center-Rizk Hospital refers to an institutional emergency response triggered by an external security threat or a mass-casualty incident, requiring lockdown procedures, mobilization of staff, and heightened security measures. The full questionnaire, including exact wording, response options, and coding scheme, is available in Appendix A to ensure full reproducibility.

Because all questionnaire items were mandatory, incomplete submissions could not be submitted; therefore, no missing data were present, and no imputation was required. The final sample consisted of 370 complete responses.

The PCL-5 is a publicly available scale used to assess the risk of PTSS development based on DSM-5 diagnostic criteria [15]. It is a self-reported, comprehensive, and validated scale in English consisting of 20 items, each rated on a 5-point Likert scale ranging from 0 (“Not at all”) to 4 (“Extremely”), yielding a total score between 0 and 80 [15]. A score of 33 or higher indicates an elevated risk of PTSS and warrants further clinical evaluation by a mental health professional [15]. Internal consistency of the PCL-5 (20 items) was excellent in the current sample (Cronbach’s α = 0.91), exceeding the commonly accepted threshold of 0.70.

Ethical considerations

The Institutional Review Board of the Lebanese American University approved both the survey and the study conduction (IRB reference: LAUMCRH.AA1.9/Dec/2024). Participation was voluntary, and informed consent was included at the beginning of the questionnaire. All responses were collected anonymously, and no identifying information was retained. Furthermore, access to the data was restricted to authorized members of the research team.

Statistical analysis

The normality of the data was assessed using the Kolmogorov-Smirnov test. Observations were assumed to be independent of each other. Given the large sample size, parametric methods were used as appropriate. Sensitivity analyses using nonparametric methods yielded similar conclusions. The independent-samples T test was used as appropriate to compare the mean of the dependent variable between two categories, and ANOVA/Welch's ANOVA or the Kruskal-Wallis test was used for more than two categories based on the differences between the variances and the sample size as appropriate. Effect sizes were reported to complement p-values and to quantify the magnitude of group differences in PCL-5 scores, given the continuous and clinically interpreted nature of the outcome. Cohen's d was reported for the t-test, eta-squared (η²) for the ANOVA test, and epsilon-squared (ε²) for the Kruskal-Wallis test. The correlation between two quantitative variables was assessed using the Pearson correlation test. The p-value < 0.05 was considered statistically significant. A multivariable linear regression model was created, and assumptions were assessed using residual plots, variance inflation factors, and the Durbin-Watson statistic, with no major violations identified. All statistical analyses were performed using IBM SPSS Statistics for Windows, Version 27.0 (IBM Corp., Armonk, NY).

## Results

The total sample size was 370 participants, with 145 (39.2%) males and 225 (60.8%) females, all Lebanese. Participants were distributed among different job profiles, mostly medical residents/fellows (196; 53%). The largest age group was 18-30 years (251; 67.8%), followed by older age groups. Most participants lived in Beirut (167; 45.1%) and Mount Lebanon (165; 44.6%). A total of 24 participants (6.5%) reported a previous history of PTSD diagnosed by a mental health provider. The results of the remaining sociodemographic and personal characteristics are presented in Table 1.

**Table 1:** Sociodemographic and personal characteristics of the participants MD: Doctor of Medicine; PTSD: post-traumatic stress disorder

Characteristics	N (%)
Gender	
Male	145 (39.2%)
Female	225 (60.8%)
Age (years)	
18 - 30	251 (67.8%)
31 - 40	50 (13.5%)
41 - 50	33 (8.9%)
51 - 64	36 (9.7%)
Marital status	
Single	170 (45.9%)
In a relationship	75 (20.3%)
Married	115 (31.1%)
Divorced/ Widowed	10 (2.7%)
Residency	
North Lebanon/ Akkar	14 (3.8%)
Beirut	167 (45.1%)
Mount Lebanon	165 (44.6%)
Beqaa	10 (2.7%)
South Lebanon/ Nabatiyeh	14 (3.8%)
Income ($)	
Below 500	10 (2.7%)
500 - 999	43 (11.6%)
1000 - 2000	263 (71.1%)
Above 2000	54 (14.6%)
Job profile	
Practical nurse	31 (8.4%)
Head nurse/Registered nurse	80 (21.6%)
Medical resident/Fellow	196 (53%)
Physician	48 (13%)
Pharmacist	9 (2.4%)
Physiotherapist	6 (1.6%)
Specialty, if MD (N = 243)	
Anesthesiology	21 (8.6%)
Dermatology	4 (1.6%)
Diagnostic radiology	16 (6.6%)
Emergency medicine	22 (9.1%)
Family medicine	12 (4.9%)
General surgery	12 (4.9%)
Internal medicine	86 (35.4%)
Neurology	10 (4.1%)
Neurosurgery	1 (0.4%)
Obstetrics and gynecology	11 (4.5%)
Ophthalmology	3 (1.2%)
Orthopedics	12 (4.9%)
ENT	4 (1.6%)
Pediatrics	15 (6.2%)
Psychiatry	7 (2.9%)
Urology	7 (2.9%)
Previous diagnosis of PTSD	
No	346 (93.5%)
Yes	24 (6.5%)

Direct effects of the war on participants included 80 (21.6%) displaced from their primary residency, 11 (3%) sustained direct injury secondary to current war events, and 59 (15.9%) experienced loss of a relative or a friend secondary to current events. The mean of PCL-5 survey scores was 18.28 ± 14.94, and 68 (18.4%) participants had a risk of developing PTSD. The results above and the remaining direct effect of the war are shown in Table 2.

**Table 2:** Direct effects of the war on the particpants and the PCL-5 score PTSD: post-traumatic stress disorder; PCL-5: PTSD Checklist for Diagnostic and Statistical Manual of Mental Disorders, Fifth Edition

Direct effects of the war	N (%) or Mean ± SD
Displaced from the place of primary residency	80 (21.6%)
Sustained a direct injury secondary to war events	11 (3%)
Relative/Friend sustained a direct injury secondary to war events	103 (27.8%)
Loss of a relative/friend secondary to the current	59 (15.9%)
Was on duty on the days a Code White was activated	226 (61.1%)
Wasn’t on duty, but presented to the hospital after Code White activation (N = 144)	93 (64.6%)
PCL-5 score	18.28 ± 14.94
Risk of developing PTSD	68 (18.4%)

Table 3 shows the difference between the PCL-5 score means of different groups. PCL-5 scores differed significantly across several subgroups, with the strongest associations observed for war-related exposures. Females reported higher PTSD symptom scores than males (p < 0.001; moderate effect). Age, marital status, and residency showed either negligible or no clinically meaningful differences despite some statistical significance. Lower income and nursing roles were associated with higher PCL-5 scores, although having small effect sizes.

**Table 3:** Difference of means of PCL-5 scores across different subgroups MD: Doctor of Medicine; PTSD: post-traumatic stress disorder; PCL-5: PTSD Checklist for Diagnostic and Statistical Manual of Mental Disorders, Fifth Edition

Groups	Mean ± std	p-value	Effect size
Gender			
Male	14.77 ± 13.45	<0.001	0.39
Female	20.53 ± 15.43		
Age (years)			
18 - 30	19.34 ± 15.65	0.015	0.02
31 - 40	17.58 ± 14.54		
41 - 50	12.88 ± 9.62		
Above 51	16.78 ± 13.49		
Marital status			
Single	18.12 ± 15.31	0.889	0.01
In a relationship	18.45 ± 16.10		
Married	17.97 ± 13.16		
Divorced/Widowed	23 ± 19.71		
Residency			
North Lebanon/ Akkar	13.93 ± 11.63	0.1	0.02
Beirut	19.34 ± 15.81		
Mount Lebanon	16.90 ± 14.10		
Beqaa	17.70 ± 12.85		
South Lebanon/ Nabatiyeh	26.57 ± 15.60		
Income ($)			
Below 500	29 ± 22.20	0.02	0.03
500 - 999	20.47 ± 13.89		
1000 - 2000	18.43 ± 15.22		
Above 2000	13.8 ± 11.13		
Job profile			
Practical nurse	20.74 ± 17.48	0.002	0.05
Head nurse/Registered nurse	23.39 ± 14.39		
Medical resident/Fellow	17.25 ± 14.82		
Physician	13.02 ± 12.92		
Pharmacist	13.11 ± 10.72		
Physiotherapist	20.67 ± 14.63		
Specialty, if MD (N = 243)			
Anesthesiology	13.1 ± 14.16	0.012	0.06
Dermatology	14.5 ± 10.66		
Diagnostic radiology	14.56 ± 9.598		
Emergency medicine	23.36 ± 17.56		
Family medicine	12.92 ± 8.67		
General surgery	17.75 ± 15.31		
Internal medicine	13.91 ± 13.53		
Neurology	20.2 ± 18.15		
Neurosurgery	13		
Obstetrics and gynecology	20.55 ± 19.49		
Ophthalmology	25.33 ± 11.50		
Orthopedics	23.25 ± 12.82		
ENT	9.25 ± 5.5		
Pediatrics	24.6 ± 14.63		
Psychiatry	9.29 ± 11.23		
Urology	5.29 ± 4.68		
Previous diagnosis of PTSD			
No	17.4 ± 14.39	<0.001	0.93
Yes	30.92 ± 17.08		
Displaced from the place of primary residency			
No	16.94 ± 14.29	0.001	0.42
Yes	23.11 ± 16.24		
Sustained a direct injury secondary to war events			
No	17.69 ± 14.47	<0.001	1.36
Yes	37.55 ± 17.67		
Relative/friend sustained a direct injury secondary to war events			
No	15.26 ± 13.62	<0.001	0.77
Yes	26.1 ± 15.41		
Loss of a relative/friend secondary to the current			
No	16.99 ± 14.27	<0.001	0.55
Yes	25.05 ± 16.59		
Was on duty the days a Code White was activated			
No	17.18 ± 14.57	0.261	0.12
Yes	18.97 ± 15.15		
Wasn’t on duty, but presented to the hospital after Code White activation (N = 144)			
No	16.67 ± 15.96	0.755	0.05
Yes	17.46 ± 13.84		

Prior PTSD diagnosis and war-related trauma demonstrated the largest effects: direct injury, displacement, and injury or loss of a relative or friend were each associated with markedly higher PCL-5 scores (all p < 0.001; moderate-to-large effects). The remaining results are shown in the table below (Table 3).

Bivariate correlations between the PCL-5 score and multiple variables are shown in Table 4. Positive and significant correlates were found between the PCL-5 score and each of female gender (r = .189, p < .001), previous diagnosis of PTSD (r = .223, p < .001), and direct effects secondary to war. However, a negative correlation was found between the PCL-5 score and higher income (r = -.164, p < .01).

**Table 4:** Bivariate correlations between the PCL-5 scores, sociodemographic characteristics, and direct war effects 1. Gender; 2. Age; 3. Marital status; 4. Residency location; 5. Income; 6. Previous diagnosis of PTSD; 7. Displacement from the location of residency; 8. Direct injury secondary to war; 9. Direct injury of a relative/friend secondary to war; 10. Loss of a relative/friend *p<0.05; **p<0.01; ***p<0.001 PTSD: post-traumatic stress disorder; PCL-5: PTSD Checklist for Diagnostic and Statistical Manual of Mental Disorders, Fifth Edition

	1	2	3	4	5	6	7	8	9	10
PCL-5 Score	.189^***^	-0.1	0.016	0.053	-.164^**^	.223^***^	.170^**^	.226^***^	.326^***^	.198^***^

In multivariable linear regression analysis, the overall model was statistically significant and explained 19.1% of the variance in PCL-5 scores (adjusted R² = 0.171, p < 0.001). Female gender was independently associated with higher PTSS severity (B = 5.71, p < 0.001). Compared with participants aged ≤30 years, those aged 41-50 years had significantly lower PCL-5 scores (B = −6.61, p = 0.010). A prior diagnosis of PTSD was associated with higher PTSS scores (B = 7.33, p = 0.037). Among war-related exposures, having a relative or friend sustain a direct injury was strongly associated with increased PTSS severity (B = 8.02, p < 0.001). Displacement, personal injury, and loss of a relative were not independently associated with PTSS after adjustment. No evidence of multicollinearity was observed (Table 5).

**Table 5:** Linear regression model for PCL-5 score Linear regression adjusted for all variables listed; Reference categories: male gender; age ≤30 years; no prior PTSD; no war-related exposure; Model R² = 0.191, adjusted R² = 0.171; F(9,360) = 9.46, p < 0.001. PTSD: post-traumatic stress disorder; PCL-5: PTSD Checklist for Diagnostic and Statistical Manual of Mental Disorders, Fifth Edition

Predictor		B (Unstandardized)	95% CI for B		p-value
			LL	UL	
Gender	Male	Reference			
	Female	5.71	2.81	8.62	<0.001
Age (years)	18 - 30	Reference			
	31 - 40	−3.64	−7.88	0.60	0.092
	41 - 50	−6.61	−11.62	−1.60	0.010
	51 - 64	−4.09	−8.91	0.72	0.096
Prior PTSD diagnosis		7.33	0.44	14.23	0.037
Displacement		1.02	−2.81	4.85	0.600
Direct personal injury		7.86	−2.10	17.82	0.121
Relative/friend injured		8.02	4.39	11.66	<0.001
Loss of a relative/friend		1.42	−3.22	6.05	0.548

## Discussion

The 2023-2024 war between Israel and Lebanon took its toll on the Lebanese people’s mental health. Yet, to date, there have been no comprehensive assessments on a nationwide level of the effect this war had on the psychological state of the Lebanese people, and particularly HCWs, a population often overlooked despite being on the front line. This study provides a critical window into this issue. It is important to state that this study was conducted at Lebanese American University Medical Center-Rizk Hospital in Achrafieh, Beirut, an area that was not directly targeted by Israeli forces. Although the hospital’s area was not directly targeted, it faced the war’s indirect repercussions: HCWs were exposed to constant threats of escalation, the sound of surrounding bombardment, and waves of displaced, injured patients. Moreover, Lebanese American University Medical Center-Rizk Hospital employs staff from different regions across Lebanon, including areas that were directly affected by the conflict. In this study, 68 participants (18.4%) were identified as being at risk of developing PTSS, emphasizing that the consequences of war extend beyond physical destruction to include a substantial psychological burden.

Overall, this study demonstrates that HCWs at a major tertiary hospital in Beirut reported varying levels of post-traumatic stress symptoms during the 2023-2024 conflict period. While the findings cannot establish causality, several associations emerged between higher PCL-5 scores and specific demographic or exposure-related factors. Female participants and nurses reported higher symptom burden in bivariate analyses, which aligns with prior literature describing increased psychological vulnerability among women and the significant emotional and physical demands placed on nurses during crises [16]. In multivariable analysis, female gender remained independently associated with higher PCL-5 scores, supporting the robustness of this finding after adjustment for potential confounders.

Additionally, not all HCWs were affected by the war equally. Across HCW job profiles, nurses, particularly head nurses and registered nurses, exhibited higher PCL-5 scores in unadjusted analyses. Nurses are among the most highly stressed workers within hospital settings, as they orchestrate much of the patient’s medical journey and are often the first point of contact for patients and their families. A previous study reported that over 70% of nurses experienced high stress levels compared with approximately 30% of physicians [17]. The war may have further amplified this stress, potentially contributing to increased PTSS, particularly given nurses’ direct involvement in the care of war casualties and the management of severe injuries [18]. However, these findings should be interpreted cautiously, as professional role was not retained as an independent factor after multivariable adjustment.

It is important to keep in mind that Lebanon has faced multiple challenges over the past decade, including a prolonged economic crisis, the 2020 Beirut Port blast, political instability, and recurring conflicts. The aftermath of such events has had a substantial psychological impact on the Lebanese population [19]. It remains unclear whether repeated exposure to adversity fostered increased resilience and a higher threshold for developing PTSD or whether the 2023-2024 war represented a cumulative stressor that further contributed to the development of PTSS.

Among physicians, ophthalmologists exhibited higher PCL-5 scores in descriptive analyses. On September 17, 2024, Israel launched an intense attack on Lebanon during which thousands of explosive pagers were detonated across Lebanon and Syria, resulting in thousands of injuries and multiple fatalities, including HCWs. Many injuries involved the face and eyes, leading to permanent visual impairment and disfigurement [20,21]. In the aftermath, ophthalmologists played a critical role in managing these injuries under extreme pressure and chaotic conditions, which may plausibly contribute to psychological distress. However, given the small size of this subgroup and the observational nature of the study, this finding should be interpreted with caution and viewed as hypothesis-generating rather than conclusive.

PCL-5 scores were significantly and negatively correlated with the income of HCWs. While lower income is broadly reflective of increased exposure to chronic stressors, mainly financial hardship, which may exacerbate acute PTSD symptoms and maintain persistent PTSD over time [22], this association did not remain statistically significant after adjustment for sociodemographic and war-related factors in the multivariable analysis. This finding is especially relevant in the Lebanese context, where inflation, currency devaluation, and loss of social security intensified the financial strain experienced by the Lebanese population in general and HCWs in particular.

Similarly, war-related events, including displacement from primary residence, direct physical injury, or the loss of a relative or friend, were significantly and positively correlated with PCL-5 scores in bivariate analyses. Individuals who were affected exhibited a notable increase in their average scores. This aligns with the well-established association between the development of PTSD and the experience of traumatic events [23]. However, in multivariable analysis, only having a relative or friend sustain a direct injury remained independently associated with higher PCL-5 scores, while displacement, personal injury, and loss of a relative were no longer statistically significant. As with all observational findings, directionality cannot be inferred due to the cross-sectional nature of the data.

Limitations

Several limitations should be acknowledged in this study. First, its cross-sectional, single-centered design provides the symptoms experienced at a specific point in time, in a specific hospital, which may limit the ability to establish causality or generalizability of the findings.

Second, the sampling approach relied on a convenience sample distributed through WhatsApp broadcast lists and departmental groups. As such, the study is susceptible to sampling bias, and the true response rate could not be determined, limiting representativeness and introducing potential nonresponse bias.

Third, the study did not measure or adjust for pre-existing or cumulative trauma exposure, including Lebanon’s recent economic collapse, the Beirut Port explosion, and the COVID-19 pandemic. These prior stressors may influence PTSS scores and act as confounders; hence, observed associations should be interpreted cautiously and cannot be inferred as independent effects.

Despite these limitations, the study successfully quantifies the toll that the war has taken on HCWs who have experienced these tragic events and supports the need for targeted mental health interventions.

## Conclusions

This study identifies elevated PTSS levels among HCWs at a major tertiary hospital in Beirut after the 2023-2024 conflict. Higher symptom burden was observed among women, nurses, lower-income staff, and individuals who experienced direct war-related adversity. These findings describe important associations but should not be interpreted as causal due to the cross-sectional design and lack of adjustment for confounding factors.

While the results may not be generalizable to all HCWs in Lebanon, they underscore the psychological strain experienced by frontline staff operating within a context of repeated national crises. These findings highlight the urgent need for targeted mental health interventions. Public health authorities have a responsibility to address the long-term consequences of repeated crises on frontline providers by implementing systematic mental health screening programs to prevent the onset of psychiatric conditions among HCWs.

## Appendices

Appendix A

Questionnaire

A. Consent

1. By selecting Yes, I willingly agree to take part in this study and confirm that I am between 18-65 years old

·       Yes

·       No

2. By selecting Yes, I confirm that I have been residing in Lebanon since the beginning of the war

·       Yes

·       No

3. By selecting Yes, I confirm that I am a member of the healthcare field (Physician, Fellow or resident, Registered nurse, Practical nurse, Pharmacist, Physiotherapist)

·       Yes

·       No

B. Sociodemographic Characteristics

4. Gender

·       Female

·       Male

5. Age

·       18 - 30

·       31 - 40

·       41 - 50

·       Above 51

6. Nationality

·       Lebanese

·       Non-Lebanese

7. Marital Status

·       Single

·       In a relationship

·       Married

·       Divorced

·       Widowed

8. Residency location prior to the current events

·       Akkar

·       North Lebanon

·       Baalbeck - Hermel

·       Beirut

·       Mount Lebanon

·       Beqaa

·       South Lebanon

·       Nabatiyeh

9. Income (Monthly)

·       Below 500$

·       500$ - 999$

·       1000$ - 2000$

·       Above 2000$

10. Job profile

·       Practical Nurse

·       Head Nurse/Registered Nurse

·       Medical Resident/Fellow

·       Physician

·       Pharmacist

·       Physiotherapist

11. If MD, which specialty?

·       Anesthesiology

·       Dermatology

·       Diagnostic Radiology

·       Emergency Medicine

·       Family Medicine

·       General Surgery

·       Internal Medicine

·       Neurology

·       Neurosurgery

·       Obstetrics and Gynecology

·       Opthalmology

·       Orthopedics

·       ENT

·       Pathology

·       Pediatrics

·       Psychiatry

·       Urology

12. Previous history of PTSD diagnosed by a mental health provider

·       Yes

·       No

C. Current Events

13. Have you been displaced from your place of primary residence secondary to current events?

·       Yes

·       No

14. Have you sustained a direct injury secondary to the current events?

·       Yes

·       No

15. Did a relative or friend sustain a direct injury secondary to the current events?

·       Yes

·       No

16. Have you experienced a loss of a relative or friend secondary to the current events?

·       Yes

·       No

17. Were you on duty at the hospital on the days a Code White was activated?

·       Yes

·       No

18. If not, did you come to the hospital on the days a Code White was activated?

·       Yes

·       No

D. PCL-5: 

The following items are from the PTSD checklist for DSM-5 (PCL-5; Weathers et al., 2013) available at https://www.ptsd.va.gov.

Below is a list of problems that people sometimes have in response to a very stressful experience. Please select for each problem the number that indicates how much you have been bothered by the problem in the past month, with each number corresponding to : 

0: not at all 

1: A little bit 

2: Moderate 

3: Quite a bit 

4: Extremely 

19. Repeated, disturbing, and unwanted memories of the stressful experience?

20. Repeated, disturbing dreams of the stressful experience?

21. Suddenly feeling or acting as if the stressful experience were actually happening again (as if you were actually back there reliving it)?

22. Feeling very upset when something reminded you of the stressful experience?

23. Having strong physical reactions when something reminded you of the stressful experience (for example, heart pounding, trouble breathing, sweating)?

24. Avoiding memories, thoughts, or feelings related to the stressful experience?

25. Avoiding external reminders of the stressful experience (for example, people, places, conversations, activities, objects, or situations)?

26. Trouble remembering important parts of the stressful experience?

27. Having strong negative beliefs about yourself, other people, or the world (for example, thoughts such as: I am bad, no one can be trusted, the world is dangerous)?

28. Blaming yourself or someone else for the stressful experience or what happened after it?

29. Having strong negative feelings such as fear, horror, anger, guilt, or shame?

30. Loss of interest in activities that you used to enjoy?

31. Feeling distant or cut off from other people?

32. Trouble experiencing positive feelings (for example, being unable to feel happiness or have loving feelings for people close to you)?

33. Irritable behavior, angry outbursts, or acting aggressively?

34. Taking too many risks or doing things that could cause you harm?

35. Being “superalert,” or watchful, or on guard?

36. Feeling jumpy or easily startled?

37. Having difficulty concentrating?

38. Trouble falling or staying asleep?
